# Vision Does Not Necessarily Stabilize the Head in Space During Continuous Postural Perturbations

**DOI:** 10.3389/fneur.2019.00748

**Published:** 2019-07-10

**Authors:** Stefania Sozzi, Antonio Nardone, Marco Schieppati

**Affiliations:** ^1^Centro Studi Attività Motorie, Istituti Clinici Scientifici Maugeri IRCCS, Pavia, Italy; ^2^Department of Clinical-Surgical, Diagnostic and Pediatric Sciences, University of Pavia, Pavia, Italy

**Keywords:** balance, vision, head stabilization, motor control, rehabilitation

## Abstract

Vision favors head stabilization in space during perturbations of standing balance. This is particularly obvious under conditions of continuous predictable perturbations as during sinusoidal antero-posterior (A-P) translations of the supporting platform. We tested here the hypothesis that under this condition the head can instead undergo large A-P oscillations, when a precision visual task is concurrently performed. We compared the head oscillations across four conditions while standing on a continuously translating platform. Eyes open (EO, no visual task), EO while reading a text fixed to the moving platform (EO-TP), EO while reading a text fixed to earth-ground (EO-TG), eyes-closed (EC). The platform translated at 0.2 and 0.6 Hz. Participants were young adult subjects, who received no particular instruction except reading the text aloud when required. Markers fixed on head, platform and text-sheet were captured by an optoelectronic device. We found that head oscillations were larger with EC than under all EO conditions. The oscillations were the least with EO and EO-TG, and intermediate with EO-TP. This was true under both low and high translation frequency, in spite of broadly smaller head oscillations at high frequency, common to all visual conditions. The distance between the head and the text was quite constant with EO-TP but fluctuated with EO-TG. The basic whole-body coordination features were moderately similar under all conditions, as assessed by the head-platform correlation coefficients and time lags. It appears that vision does not produce head stabilization in space when a concurrent visual task requiring focusing on a reading-text moving with the platform is performed. Contrary to traditional views centered on the stabilizing effect of vision under both static and dynamic conditions, the results show that head stabilization, normally ensuring a reference for inertial guidance for body balance, can be revoked by the CNS to allow performance of a non-postural task. This novel paradigm can shift long-standing views on the effect of vision on equilibrium control and be considered a potential exercise treatment for enhancing the multisensory integration process in people with balance problems.

## Introduction

When a person balances on a platform that continuously moves fore and aft in a predictable way, vision definitely helps reduce head oscillations, to a different extent depending on the frequency of the translations (1, 2). With eyes open, subjects tend to stabilize their head in space while the lower limbs move with the platform, so that the movement of the body resembles that of a pendulum. Closing the eyes changes the balancing pattern from “head-fixed-in-space” to “head-riding-with-the-platform” (or even oscillating more than the platform), therefore body motion approaches that of an inverted pendulum. Previous studies showed that these two different behaviors are consistent across conditions and subjects, are automatically produced by the subjects as soon as the platform starts to move under a given visual condition, are robust to proprioceptive disturbance (3), and are put in action almost immediately after a change in the visual conditions during a series of continuous perturbation cycles (4).

The head-fixed-in-space pattern has been attributed to the stabilizing effect of vision [see (5–9)]. Since decreased head and body sway with eyes-open (EO) compared to eyes-closed (EC) is observed both under static and dynamic conditions, reduced head oscillations are generally interpreted as the necessary result of the integration of the visual information into the neural processes responsible for the maintenance of equilibrium (10–13). This behavior has been observed in response to haptic information (14, 15) or information from the proprioceptors (16–21) and vestibular apparatus (22–24). This interpretation is also supported by the progressively increasing head oscillations as a function of decrease in visual acuity while standing on the continuously translating platform (25). That study showed that head stabilization in space decreases gradually when visual acuity levels are progressively reduced by adding *ad-hoc* test lenses, to reach the oscillation level of the EC condition when acuity fell below 0.001/10.

A different explanation would be that, since vision provides significant information from the environment, the visual input is valued by our brain independently of any posture-stabilizing function, but rather as function of the concurrent visual task at hand (26). If the visual information from a visual target continuously moving with the body's support base captures attention, head wavering with respect to the visual target would be kept to a minimum as a strategy allowing for clear vision, rather than as part of the body-stabilizing strategy. This would occur at the expense of ample head oscillation in space, renouncing to the head-fixed-in-space strategy.

In this line, one would also consider that the vision-induced head stabilization while standing on the continuously translating platform may not inevitably confer a strong advantage to the overall control of body balance. Indeed, both the eyes-open and the eyes-closed body oscillation patterns are equally “safe.” Normal healthy subjects easily comply with the continuous perturbation and very rarely make a step to counteract the disturbing effects of the moving platform, and never fall over, regardless of the balancing task being performed under either eyes-open or eyes-closed condition, or regardless of the subjects' age (27, 28). Further, the extent of back-and-forth displacement of the center of mass is similar between EC and EO perturbations (28–30). Moreover, blind subjects balance on the moving platform like sighted subjects eyes-closed do, be the blindness acquired or congenital (31). Although, in principle, one would have argued that blind subjects might have learned to optimally exploit their intact proprioception and the gravito-inertial inputs in order to diminish the head oscillation similarly to sighted subjects so as to stabilize their body and protect themselves from falling (32, 33). Therefore, vision does not seem to confer any particular stability under conditions of continuous predictable balance perturbations.

Hence, we set out here to test the alternative hypothesis that the head can largely oscillate even with eyes-open, when a vision-dependent concurrent task can be performed successfully through large head oscillations. To this aim, we simply compared the head oscillations eyes-open across three conditions. These consisted in (1) free watching the laboratory space (EO) or concentrating on a visual target. In turn, (2) the target was moving jointly with the platform upon which the subject stood (EO-TP), or (3) it was immobile in space, fixed with respect to the environment and not moving with the platform (EO-TG). The target consisted in a text that subjects would read aloud, in order to ensure that gaze was focused on the target and did not wander from text to environment. In the case of target on moving platform (EO-TP), the subject would allow a sizeable head oscillation in order to keep the distance between the eyes and the text within a small range. When the target was fixed to the ground (EO-TG), their head would oscillate less, again in order to keep a fairly constant distance between the eyes and the text, now stationary with respect to the moving platform.

If the hypothesis would be confirmed, head stabilization in space would not be necessarily equivalent to “better” balance. The flexibility of coordination modes (34) and its complying with non-postural tasks would speak for the capacity of the nervous system to adaptively modulate the weight attributed to balance-relevant sensory inputs. On the translational side, since continuous movement of the supporting platform, with eyes-open or eyes-closed, has been exploited as a rehabilitation procedure for various populations with balance disorders (24, 27, 35, 36), adding to the training protocol visual tasks that challenge the visual integration process may offer an additional tool for understanding and treating these disorders.

## Methods

### Subjects

Twenty (10 males and 10 females) healthy young subjects volunteered to participate in this study. Their mean age, height, and weight were: 27.7 years ± 6.5 SD, 173.8 cm ± 9.2 SD, and 66.1 kg ± 10.7 SD. Subjects were naïve to the experimental procedures and all succeeded in performing the trials. All recruited subjects had normal vision (corrected by eyeglasses or corneal lenses in 8 participants). Subjects gave written informed consent to participate in the experiments, which were performed in accordance with the Declaration of Helsinki. The institutional ethics committee (Ethics Committee, Istituti Clinici Scientifici Maugeri IRCCS) specifically approved the study (number # 2257 CE).

### Task and Procedures

Subjects stood with bare feet spaced about 10 cm apart and with the arms by their side on a mobile platform (Officina Lomazzi, Italy), translating horizontally in a sinusoidal way in the antero-posterior (A-P) direction at a constant frequency of approx. 0.2 Hz (nominal value 0.18 Hz) and 0.6 Hz (nominal value 0.56 Hz) in different trials and with a constant amplitude of 10 cm (for both frequencies). Subjects performed one try-out (per translation frequency, both with eyes-open and with eyes-closed) in order to familiarize with the platform movement, then the experiment started. Each acquisition epoch lasted about 3 min for 0.2 Hz or about 1 min for 0.6 Hz platform translation frequency. Each epoch was composed of a period of at least 5 s in which subjects stood quietly on the still platform, then the platform started to move and delivered 27 and 31 consecutive oscillation cycles at low and high frequency, respectively. Subjects performed a series of trials under four different conditions, standing at the center of the platform: (1) eyes open (EO) during which they were simply looking at the structured laboratory environment in front of them; (2) reading a text printed on a A4 sheet (normally spaced words, 13-point black Arial font, 33 horizontal lines spaced by 1.5 lines, aligned left) fixed in space at eye level and distant about 50 cm from the forehead (eyes-open, text-on-ground, EO-TG); (3) reading a text at the same eye level as above, the text now moving with the platform (eyes-open, text-on-platform, EO-TP). In this condition, the support of the text was fixed at the base of the translating platform, again distant about 50 cm from the forehead; (4) eyes closed (EC). Each condition was repeated two times at each oscillation frequency and vision condition (amounting to 16 trials for each subjects). The trials were randomized across subjects and conditions. During the trials in which the subjects were reading the text, they were asked to begin reading aloud at a start signal given by the operator, just before the platform started to move, and to continue reading the text until the platform stopped. The narrative content of the text changed from trial to trial. All subjects were efficient readers, read fluently and had a good comprehension of the content, based on answers to questions asked at the end of each trial. Of note, no other instruction was given the participants about posture; in particular, nothing was mentioned about whether and how to counteract the perturbations or about the position of the head in space. Neither were participants asked to move the head so as to deliberately follow the motion of the visual target in the antero-posterior plane and maintain a constant distance between target and head. The trials at different visual conditions and translation frequencies were separated by a time-period from 2 to 5 min, during which subjects were free to move and rest. The entire experiment lasted <2 h, including the resting period.

### Data Recording and Analysis

Kinematic data were acquired by means of an optoelectronic device (Smart-D, BTS, Italy). In order to record the head displacement, five reflective markers were placed in the following positions: vertex, bilaterally on the lateral head and on the forehead by means of a helmet and on the 7th cervical vertebra (C7). Additionally, one marker was placed on the platform to record the platform movement and one marker was placed on the text page in order to measure the distance between the text and the head (forehead marker) during the balancing trials. The markers' position in space was recorded by 12 infrared cameras (BTS) at a sampling frequency of 140 Hz and stored in a PC for offline analysis.

For each subject, the peak-to-peak (P-P) horizontal head displacement during the platform movement was calculated from the A-P displacement of the marker placed on the vertex for each translation cycle, trial and condition. The mean P-P displacement was computed for all perturbation cycles across the entire duration of the trials and considered as an index of the A-P oscillation of the head in space.

For each subject and condition, a cross-correlation (CC) analysis was performed between the traces of the platform and of the vertex A-P displacements, in order to assess the degree of coupling between motion of the platform and motion of the head. The CC coefficient (R) at time lag = 0 was calculated by a software developed in Labview (National Instruments, Austin, TX, USA). A positive value of the CC coefficient indicated in-phase displacement of head and platform, a negative coefficient anti-phase displacement. The time lag was also computed. This was the time interval at which the absolute value of R was maximum. A positive time lag indicated a delay of the head with respect to the platform movement. The accuracy of the time-lag estimation was of 7.1 ms, according to the sampling frequency of 140 Hz.

The head pitch inclination angle was computed as the angle between the segment defined by the vertex-C7 markers and the vertical, as a proxy of neck and head position in the sagittal plane. Since the head frame was repositioned at each trial, the mean pitch angle was referred to the pitch angle recorded while subjects were standing quietly just before the onset of the perturbation cycles.

### Statistical Analysis

Two-way repeated-measure ANOVA with translation frequency (0.2 and 0.6 Hz) as factor and visual conditions (EO, EO-TP, EO-TG, EC) as repeated-measure was used to compare: head peak-to-peak displacement, the CC coefficient after z-transformation, time-lags between head and platform and head pitch inclination. Variations in head-text distance between EO-TP and EO-TG were also analyzed with repeated-measure ANOVA with translation frequency as factor. For all ANOVAs, the *post-hoc* test analyses were made with Tukey HSD test. The software package was Statistica (StatSoft, USA).

## Results

### Head Antero-Posterior Oscillation (Head Peak-to-Peak Displacement)

**Figure 1:**
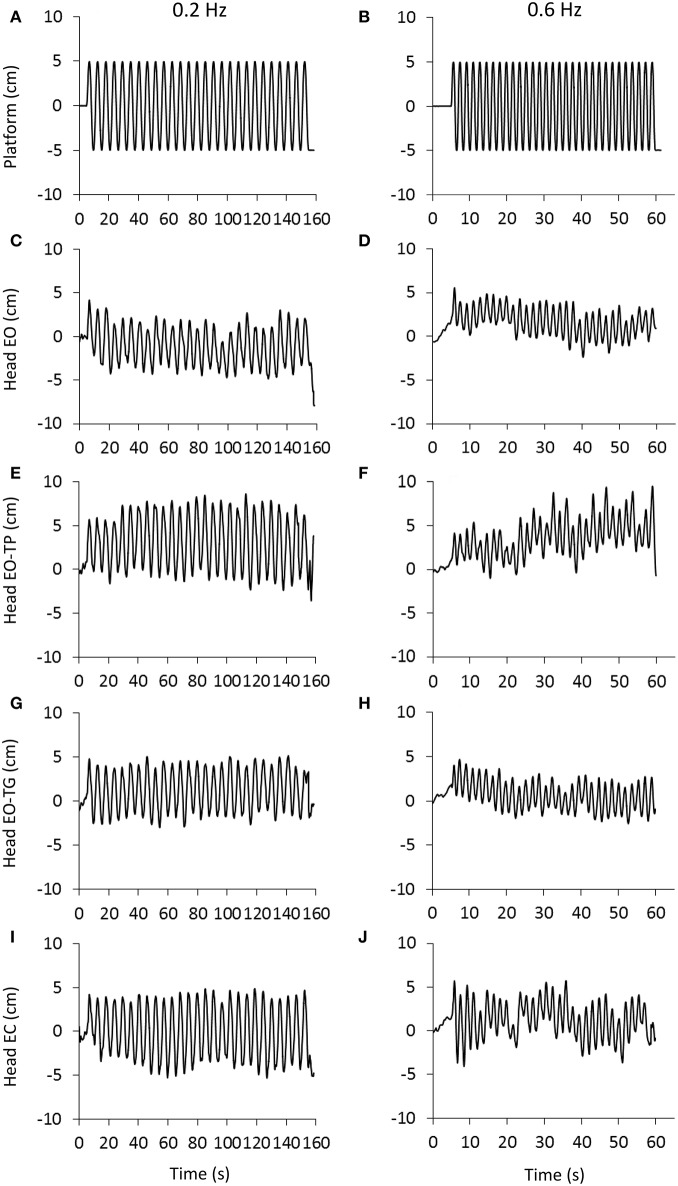
Head displacement under the different visual and perturbation conditions. **(A,B)**, platform translation at 0.2 Hz (left) and 0.6 Hz (right).The platform started to move at t = 5 s and delivered 27 **(A)** and 31 **(B)** consecutive translation cycles with a constant amplitude of 10 cm. **(C–J)**: head displacement (marker placed on the head vertex) recorded in a representative subject under eyes open condition (EO, **C,D**), reading a text printed on a sheet moving with the platform (EO-TP, **E,F**), reading a text fixed to the ground (EO-TG, **G,H**) and under eyes-closed condition (EC, **I,J**). Note the difference in time scale between the columns. Increasing values in the ordinate indicate forward movement.

Figure 1 shows the traces of the head peak-to-peak (P-P) antero-posterior (A-P) displacements recorded in a representative subject. The traces for the 0.2 and 0.6 Hz platform translation frequencies are represented in the left and right columns, respectively. The rows are the visual conditions: eyes-open (EO, Figures 1C,D), EO text on platform (EO-TP, Figures 1E,F), EO text on ground (EO-TG, Figures 1G,H), eyes-closed (EC, Figures 1I,J). Under all conditions, the head broadly moved together with the platform, the amplitude of the P-P displacement being definitely smaller EO and EO-TG, and larger with EO-TP and EC.

**Figure 2:**
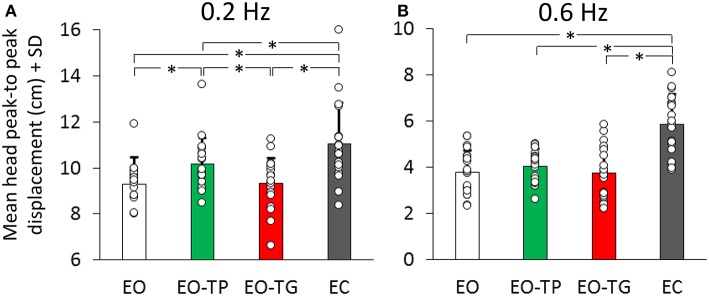
Mean head peak-to peak (P-P) displacement at 0.2 Hz **(A)** and at 0.6 Hz **(B)** translation frequency under the four visual conditions (EO: white bars, EO-TP: green bars, EO-TG: red bars and EC: gray bars). Each white dot superimposed to the bars corresponds to a subject. There was a significant difference in the mean P-P head oscillation across visual conditions. With EC, head P-P was the largest, while EO and EO-TG were the conditions in which the head P-P was the smallest. With EO-TP, head displacement was intermediate between EO and EC. Note the ordinate shift between the panels. In this and in the following Figures, the asterisk indicates a significant difference.

The average values of the head oscillation in the whole population, under the two frequency and four visual conditions are reported in Figure 2. Two-way analyses of variance showed a difference in the P-P displacement values between visual conditions [*F*_(3, 57)_ = 83.59, *p* < 0.001] and between translation frequencies [*F*_(1, 19)_ = 385.28, *p* < 0.001]. There was an interaction between frequency and visual condition [*F*_(3, 57)_ = 3.42, *p* < 0.05].

At 0.2 Hz (Figure 2A), the *post-hoc* test indicated that the head P-P was the largest with EC (*p* < 0.01, for each comparison with the three eyes-open conditions). With EO-TP, head displacement was larger than under both EO and EO-TG conditions (*p* < 0.01 for both comparisons). No difference was found between the EO and EO-TG conditions (*p* = 0.99).

At 0.6 Hz (Figure 2B) a similar picture emerged. However, not all differences reached significance. Again, head displacement in each of the three eyes-open conditions was smaller than that at EC (*p* < 0.001, for all three comparisons). Head displacement EO-TP was not significantly larger than EO (*p* = 0.89) and EO-TG (*p* = 0.83). There was no difference between the EO and EO-TG conditions (*p* = 1.0).

**Figure 3:**
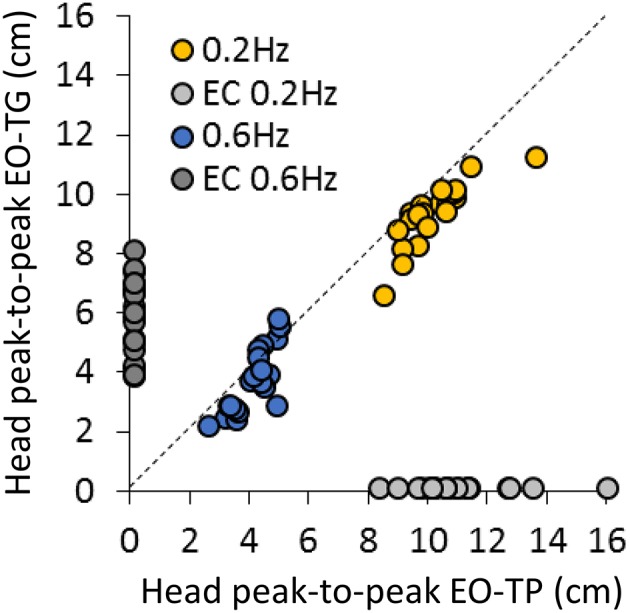
Comparison of head P-P displacements between the EO-TP and the EO-TG condition at 0.2 Hz (yellow dots) and at 0.6 Hz (blue dots) translation frequency. Each point corresponds to a subject. The dotted line is the identity line. Light gray and dark gray dots correspond to the head P-P under EC condition at 0.2 Hz (near the abscissa) and 0.6 Hz frequency (near the ordinate), respectively. At 0.2 Hz, P-P values were always smaller at EO-TP than at EO-TG. A similar but less consistent behavior was observed at 0.6 Hz.

Figure 3 concisely compares the P-P displacement of the head for all subjects under the two EO-conditions of interest, i.e., EO-TG and EO-TP, and for both platform translation frequencies. The plots show the data points of all subjects, one set (EO-TG) plotted against the other (EO-TP). Obviously, the head displacement in space was smaller EO-TG than EO-TP in all subjects at 0.2 Hz (regression line, not shown in Figure: y = 0.81 × +1.11, R^2^ = 0.71, *p* < 0.001). This was not always so for a subset of subjects at 0.6 Hz (y = 1.29 × −1.57, R^2^ = 0.64, *p* < 0.001). For reference, the corresponding P-P displacements EC at 0.2 Hz and at 0.6 Hz are reported close to the abscissa and ordinate, respectively.

### Distance Head-Text Under the EO-TP and EO-TG Conditions During Platform Displacements

**Figure 4:**
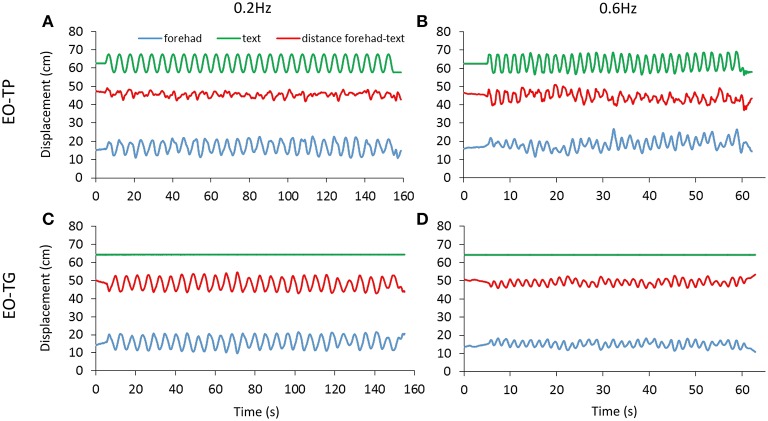
Head-text distance. The distance (red trace) between the marker on the forehead (blue trace) and the marker on the text page (green trace) was measured under EO-TP **(A,B)** and EO-TG **(C,D)** conditions for both translation frequencies (0.2 Hz left, 0.6 Hz right). The traces of a representative subject are reported. The mean distance between head and text was about 50 cm under both conditions. Increasing values in the ordinate for the forehead and text displacement traces indicate forward movement.

The distance between the marker on the forehead and that on the text sheet was measured for both EO-TP and EO-TG conditions. Figure 4 shows the traces of the markers on the head and text page during the A-P platform oscillations in a representative subject. Across subjects and conditions, the mean head-text distances (red) varied between 41 and 53 cm, with wide overlapping across subjects and conditions.

**Figure 5:**
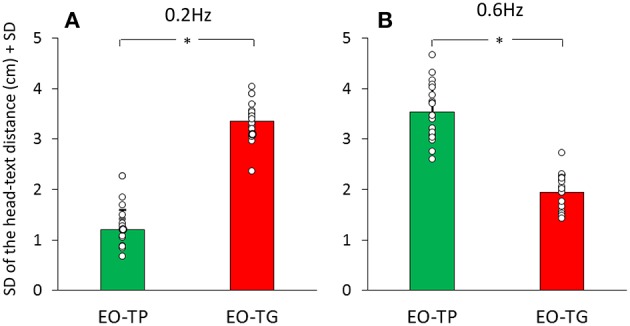
Mean standard deviation of the head-text distance at 0.2 Hz **(A)** and 0.6 Hz **(B)** translation frequency. Green bars correspond to the EO-TP condition, red bars to the EO-TG condition. The white dots superimposed to the bars correspond to the standard deviation of the head-text distance of all subjects. *Indicates a significant difference.

Figures 5A,B give an indication of the variability in these head-text distances, estimated by the average of the standard deviations of the mean traces calculated for each subject and for the two text-reading conditions. There was a significant difference between translation frequencies [*F*_(1, 19)_ = 46.54, *p* < 0.001] and between visual conditions [EO-TP vs. EO-TG: *F*_(1, 19)_ = 12.6, *p* < 0.01]. The interaction between translation frequencies and visual conditions was significant [*F*_(1, 19)_ = 386.82, *p* < 0.001].

At 0.2 Hz, the head-text distance under the EO-TP condition (mean distance = 46 cm) remained remarkably constant (small standard deviation) during the platform translations, because the head moved with the platform and text. The head-text distance varied more under the EO-TG condition (*p* < 0.01).

At 0.6 Hz, this picture changed somewhat, the head-text distance at EO-TP (mean distance = 45 cm) being less constant than at 0.2 Hz. At 0.6 Hz, the head-text distance (mean distance = 46 cm) was less constant under EO-TP than EO-TG (*p* < 0.001). In the latter case (EO-TG), the head stabilizing effect of the fixed-in-space target text was more obvious than in the 0.2 Hz condition.

### Time Relationship Between Head and Platform Displacement

**Figure 6:**
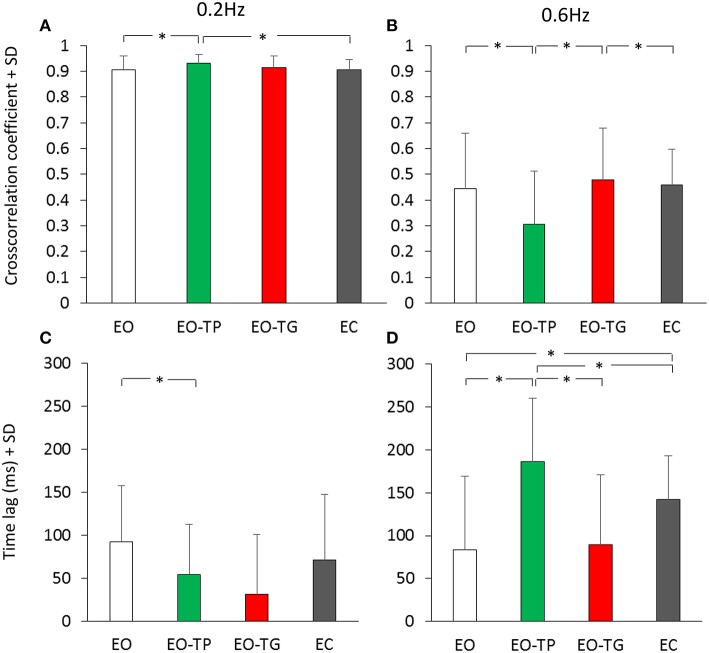
Cross-correlation between head and platform displacement. **(A,B)** mean cross-correlation coefficient (CC) calculated for the 0.2 Hz **(A)** and 0.6 Hz **(B)** translation frequency. **(C,D)** time-lag between head and platform displacement at 0.2 Hz **(C)** and 0.6 Hz **(D)** translation frequency. White bars refer to the EO condition, green bars to the EO-TP condition, red bars to the EO-TG condition and gray bars to the EC condition. The CC coefficients were positive under all visual conditions, indicating in-phase movement of head and platform, and were greater at 0.2 Hz than at 0.6 Hz translation frequency. Mean time-lags were positive under all visual and frequency conditions indicating that the head displacement followed the platform displacement. *Indicates a significant difference.

The effect of vision and of the text-reading conditions on the cross-correlation between head and platform displacement have been assessed. Figure 6 shows that the average value of the cross-correlation function for all conditions was high and low at 0.2 Hz (Figure 6A) and 0.6 Hz (Figure 6B) platform translation frequency, respectively. There was a significant difference in the CC values between translation frequencies [*F*_(1, 19)_ = 593.85, *p* < 0.001]. Subjects preferably rode the platform at low frequency, while they were less successful in counteracting the platform perturbations while attempting to remain in equilibrium at high frequency. ANOVA across visual conditions was not significant [*F*_(3, 57)_ = 1.09, *p* = 0.36]. There was a significant interaction between translation frequencies and visual conditions [*F*_(3, 57)_ = 16.14, *p* < 0.001]. At 0.2 Hz, the CC value under the EO-TP condition was the largest and significantly different from the EO and EC condition (*post-hoc, p* < 0.05 for both comparisons) but not different from the value at EO-TG (*p* = 0.23). At 0.6 Hz, the CC value for EO-TP was the smallest across all conditions (*p* < 0.01, for all comparisons).

The average time-lags between head and platform displacements were regularly positive (head following the platform) under all conditions (Figures 6C,D), but the lag values were small and non-significantly different from zero in all subjects (37). On average, there was a significant difference in the mean time-lags between translation frequencies [*F*_(1, 19)_ = 11.33, *p* < 0.01] because at 0.6 Hz the time-lags tended to be larger than at 0.2 Hz. ANOVA between visual conditions was significant [*F*_(3, 57)_ = 10.84, *p* < 0.001]. There was a significant interaction between translation frequencies and visual conditions [*F*_(3, 57)_ = 12.28, *p* < 0.001]. At 0.6 Hz, the largest time-lag was observed for the condition EO-TP (EO-TP > EO and EO-TG, *p* < 0.001).

### Head Pitch Inclination During EO-TP and EO-TG Trials

**Figure 7:**
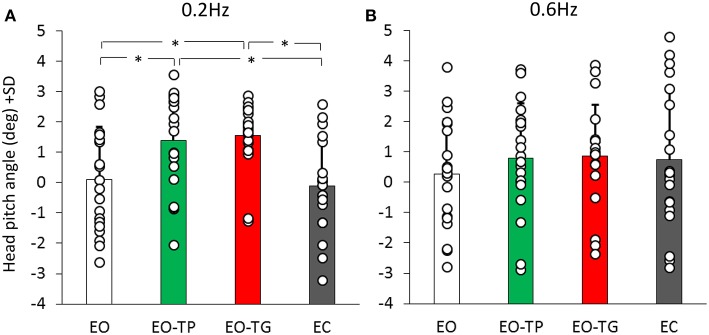
Head pitch inclination at the 0.2 Hz **(A)** and 0.6 Hz **(B)** translation frequencies (positive values mean head forward pitch). White bars refer to EO condition, green bars to EO-TP condition, red bars to EO-TG condition and gray bars to EC condition. Each white dot superimposed to the bars corresponds to the head pitch inclination of one subject. There was no difference in the head pitch inclination in the two text-reading conditions for both translation frequencies. *Indicates a significant difference.

Since head position in the sagittal plane might affect the balancing behavior through neck or labyrinth input, head pitch angle was used to estimate neck and otolith receptor activation between conditions. Figure 7 shows the mean head pitch angle for both platform translation frequencies and for the different visual conditions. ANOVA showed no difference between translation frequencies [*F*_(1, 19)_ = 0.03, *p* = 0.86], but a significant difference between visual conditions [*F*_(3, 57)_ = 5.19, *p* < 0.01]. There was a significant interaction between translation frequencies and visual conditions [*F*_(3, 57)_ = 4.02, *p* < 0.05].

Notably, the head pitch angle was similar under the two text-reading conditions at 0.2 Hz (*post-hoc, p* = 0.99). A small difference of about 1 deg was present between the text-reading conditions and both EO (*p* < 0.05 for both EO-TP and EO-TG) and EC conditions (*p* < 0.01 for both EO-TP and EO-TG). At 0.6 Hz the picture was similar, but no statistical difference was found between the different visual conditions (*p* > 0.7 for all comparisons).

## Discussion

Manipulation of vision has been mostly performed under standing conditions on ground while performing tasks of different nature (26, 30, 38–44). Head stabilization with vision, compared to oscillations eyes-closed, has been repeatedly observed in subjects standing on a continuously moving base of support as well (see section Introduction). Whether or not head stabilization can be renounced as needed under this critical balance condition, even in the presence of full vision, has never been considered.

We have confirmed here that the head oscillations of standing subjects during a series of sinusoidal balance displacements in the antero-posterior direction are definitely large with eyes closed (EC) and small with eyes open (EO). This is in keeping with numerous original reports in the literature and in review papers (45). In the present study, the peak-to-peak (P-P) head displacement with EC was larger than the amplitude of the platform displacement, and larger than head P-P displacement in the three different EO conditions tested. However, important differences existed within the EO conditions. The head oscillations were larger when subjects were reading a text that moved with the platform upon which they stood (EO-TP), compared to when subjects read a text stationary in space (fixed to ground, EO-TG) or to when they were simply looking freely at the laboratory wall in front of them (EO, no text reading).

These conclusions hold true for both platform oscillation frequencies (0.2 and 0.6 Hz), even if head oscillations were overall smaller and more variable at high than low frequency. In both cases, beyond the known differences in the balancing pattern (46), the head oscillated more when the text was fixed to the translating platform and moved with it compared with when the text was stationary, fixed to the firm ground. Hence, a particular visual task can take priority over postural stabilization and let head oscillations increase to allow for accomplishment of the visual task. However, when a more challenging postural perturbation has to be counteracted during the visual task (as at high translation frequency), in spite of a grossly similar behavior across visual condition, head oscillations are overall reduced. This suggests that balance control is adjusted to maximize stability when posture is challenged but can be adjusted to accommodate a “cortically important” task if doing so does not compromise postural stability. For simplicity, the discussion below deals with the 0.2 Hz condition. The discussion of the differences between oscillation frequency conditions will follow next.

### Low Frequency Platform Translations

#### Text on Platform

When the subjects were reading the text moving with the platform (EO-TP), the head oscillation range was the greatest among the three eyes-open conditions. The head oscillation had an overall peak-to-peak (P-P) amplitude slightly larger than that of the platform displacement, and intermediate between the oscillations under the EO and EC conditions. Further, the distance between the head and the text sheet was almost invariant across the EO-TP perturbation cycles. The mean cross-correlation (CC) between the traces of the head antero-posterior translations and of the moving platform (to which the text was fastened) was close to 1, and the mean time lag between the displacements of the markers fixed to the head and to the text was very small, i.e., the head moved almost *en-block* with platform and text. Hence, under this condition, subjects did abandon the head-fixed-in-space pattern, described in previous investigations as a hallmark of the eyes-open balancing behavior when standing on a continuously translating platform (2, 31). Subjects prioritized the invariant distance between the head and the target text, by anchoring the head to the text moving with platform and body, a task that was easily accomplished.

#### Text on Ground (Fixed With the Environment)

With the text fixed to the still ground (EO-TG), the head was kept more stationary in space than under the EO-TP and EC conditions. The peak-to-peak amplitude of head oscillation was significantly smaller than that with text-on-platform (EO-TP) discussed above, and similar to that with eyes open (EO, no text reading). Hence, with the reading-text fixed with respect to the environment, the head behavior corresponded to that described elsewhere as “head-fixed-in-space.” The mean distance between head and text (text-on-ground) had a standard deviation larger than with EO-TP, though. In other words, the mean distance was not different between the two conditions, but the variation around this value was larger for EO-TG because the head was not fully anchored to the text. The correlation between head and moving platform was high but significantly smaller than that of the correlation between the same variables in the condition text-on-platform (EO-TP). Thus, under both EO-TP and EO-TG conditions the basic relationship between head and platform periodic displacements was maintained, but with EO-TP mastering the head-text distance was prioritized with respect to body segments' coordination.

We had anticipated that the head displacement under the condition EO-TG could have been smaller than actually found, because subjects' attention was focussed on the text and subjects had a fixed visual target, much as fixing a close stationary spot reduces the body sway when standing quietly (47, 48). It is arguable, though, that in the present case, the observed behavior was the best trade-off between clear reading and the balance constraints connected with the platform continuous translations. Further, restricting the head displacement around a fixed position in space, while the body is being continuously displaced with the platform, would not be a simple task, and would require an additional effort connected with precise anticipation and fine control of neck and trunk muscles in order to allow clear reading.

### High-Frequency Platform Translations

The 0.6 Hz frequency was also tested in this experimental paradigm, because we wanted to check our initial hypothesis (vision does not necessarily stabilize the head) against a more challenging balancing condition, where somatosensory cues would provide inaccurate orientation information. This would result from the great variability in the balancing behavior within subjects and trials at this high platform translation frequency (46) that would prevent to reliably exploit the proprioceptive input, either for producing adapted reflex responses or for creating appropriate anticipatory balance-correcting responses. Of note, the P-P head displacement was overall much smaller at 0.6 Hz than at 0.2 Hz, under all visual conditions. This was likely due to body inertia and faster velocity of the antero-posterior displacement of the supporting platform that prevented “riding” the platform. High bandwidth perturbations have been shown to minimize head-in-space translation (49), also because of the stronger efforts necessary to counteract the balance challenge. This behavior (reduced head A-P oscillations) has been repeatedly observed (1, 2, 17, 28, 50) and is confirmed here.

In spite of the generally smaller head oscillations with eyes open at high than low frequency, again we observed at 0.6 Hz small P-P head displacements with text-fixed on earth (EO-TG) and large head displacements with text-on-platform (EO-TP). At 0.6 Hz in the EO-TP condition, the mean standard deviation of the head-text distance was somewhat larger than at 0.2 Hz, meaning that the head could not be held at a quasi-constant distance from the moving text when the translation of the support base was fast. This is associated with a low cross-correlation value (see Figures 6A,B) and a large time-lag between head and platform traces, expressions of a difficult body segments' coordination and head synchronization with platform and text movement. Conversely, in the text-on-ground (EO-TG) condition, the standard deviation of the head-text distance was smaller than in the same visual condition at 0.2 Hz. It is plausible that, under the EO-TG, 0.6 Hz, the fixed text became a useful visual anchor for counteracting the challenging perturbations. On the other hand, the moving text (EO-TP) would not serve as an anchor during high frequency perturbations, as if, without an earth-fixed reference, subjects tended to prioritize balance control more than the constant head-text distance.

### Central and Peripheral Visual Flow Under the EO-TP and EO-TG Conditions

The interior of the laboratory was a stationary visual space reference under all conditions. Under the text-on-platform condition (EO-TP), at least at 0.2 Hz where the head oscillates to a large extent, the head-text distance changes very little, as discussed. The foveal input is arguably very steady, but the peripheral retinal input is changing more than under any other eyes-open condition due to the large head oscillation in space. Conversely, when the text is fixed on earth (EO-TG), head oscillations are smaller than with EO-TP, but the head-text distance is varying more, because the head is not really “stabilized in space.” This would entrain a moderate radial visual flow affecting both the fovea and the periphery of the visual field. The visual input from the periphery (including information from the structured environment) should have exerted a balance stabilizing effect, because peripheral vision is highly sensitive to movement in the environment (51–53), and should have contributed to a relatively good automatic head stabilization (54, 55). On the other hand, the changes in the peripheral visual field could be considered as a moving visual scene stimulus, and as such could have potentially entrained an antero-posterior body sway, as in Oullier et al. (42). However, in our protocol the support base moved with respect to a fixed environment, so that visual-vestibular conflict was avoided, while in Oullier et al. (42) and van Asten et al. (56) the subjects were standing still during the visual scene displacement. Both support surface translation and moving visual scene had been tested by Keshner et al. (57), who found that head oscillation increased in amplitude when both inputs were presented coincidentally. In spite of obvious differences in the protocols, their finding would support the possibility that the head oscillation can be affected by vision also during critical balance conditions.

It would be no wonder that the visual input from the peripheral retina be modulated by the task at hand (58). In the EO-TP case, at low translation frequency, the changing peripheral visual input would cooperate with the gravito-inertial input in allowing the head to move concomitantly with the moving text and reduce the expansion/contraction of the foveal field. In this condition, the gravito-inertial input would be large as well (5), due to the large head displacement entraining high head accelerations at the platform turning points. Conversely, under the EO-TG condition, the head displacements with respect to both the fixed text (and the environment) would be associated with an expansion/contraction of the entire visual field. Under static conditions, this would counteract head stabilization in the same way as the effect induced on postural sway by sinusoidally expanding and contracting optic flow in quietly standing subjects (7). Under our dynamic condition, the changes in the peripheral visual flow would be interpreted by the postural control system like the similar changes occurring during the EO (no task) condition. All in all, our findings are difficult to reconcile with the view that the vision necessarily helps stabilize the head “in space.” If anything, peripheral vision allows a large head oscillation when the head-text distance is constant (EO-TP, when the foveal flow is unchanging) by calibrating the anticipatory adjustments. Conversely, head is stabilized in space when both foveal and peripheral field are expanding and contracting.

### Head Stabilization in Space vs. Balance Control

Thus, we are left with the conclusion that, when a subject focuses on the text fixed to the moving platform at about 50 cm distance, thus moving with the platform at the very same frequency and displacement range, the head moves with the platform and text. The head oscillation is larger than under the condition in which subjects read an immobile text fixed on ground, or when subjects simply look at the environment. Therefore, vision availability *per se* does not necessarily produce the least possible head displacement. Head can be easily allowed to oscillate when this represents an advantage for the task at hand.

As a corollary, head and body stabilization in space during continuous perturbations of balance is not an automatic outcome of vision availability. If this were so, head stabilization in space would be a strong attractor, but this is obviously not a general rule. For instance, functional brain plasticity in blind subjects over years (31, 59), or continuous administration of eyes-closed perturbations in sighted people for an extended period of time (28), would have prompted a reduction of the head oscillations, but this does not occur. Therefore, the present findings prompt a reconsideration of the stabilizing effects of vision. Vision stabilizes the head in space if this is functional to the exploration of the environment or to reading a text substantial with the environment itself. When the text moves along with platform and body, the head is set free to move, with the aim of keeping the head-text distance within restricted limits, convenient for clear vision.

### Reweighting of Proprioceptive and Labyrinthine Input by the Concurrent Visual Task

Spindle input from various muscles, as produced by vibratory stimulation, has no striking effect on the balancing behavior under the balance-challenging conditions described here, according to De Nunzio et al. (3). Such input would be likely canceled because it is hardly reliable due to the complex and continuously changing coordination pattern of distant muscles, particularly at the higher platform oscillation frequency. Or, proprioception would be down-weighted under these conditions, in which anticipatory postural adaptations seem to play a major role (28, 29, 60). Impaired proprioception, on the other hand, does not severely affect the balancing behavior on the oscillating platform, even when it affects body sway at rest (19–21). By analogy, impaired labyrinthine input is easily tolerated when balancing on the translating platform (24). The gravito-inertial input is behaviourally dependent at the level of the vestibular nuclei (61), and the brain can modulate the vestibular-motor input-output functional maps quickly and reorganize the balance responses to compensate for vestibular disturbance (62).

Vestibular-evoked muscle activity is a highly flexible response organized to compensate for a particular balance task (63–65). The particular task at hand here would certainly activate the vestibular otolithic receptors, and these would contribute in a task-dependent manner in the modulation of the activity of the neck and trunk muscle (66). Otolith receptors would probably not be activated in a strikingly different way under the two text-reading balancing conditions studied here, because the head pitch angle was similar. If anything, we would note that visual fixation, which normally improves head stabilization in space in cooperation with the vestibular input (67), did *not* stabilize the head under the EO-TP condition, as discussed above. As a further consideration, we would add that proprioceptors and gravito-inertial receptors seem not to counteract, but rather assist the head-moving-with-platform behavior, despite vision availability. It is known that visual and vestibular cues are fused to provide estimates of head orientation and velocity in space (68). We are not in the position of suggesting a way this fusion occurs, but certainly these inputs do not force a “set” head oscillation range independent of the visual task. Further research would be needed to test this proposition.

The above findings highlight the critical role of the anticipatory processes as key players in the control of dynamic balance when the perturbation is predictable (28). Anticipatory adjustments would be quite rightly adapted to the task at hand: maintaining clear vision of a target of interest. In a sense, this is exactly their function. They care that our head does not hit a door when we are to open it, and perfectly adapt the force of the leg muscles to the force of the forearm muscles necessary to open the door (69, 70). No wonder they would be able to adjust the trunk and head oscillations to the visual task at hand, particularly under the present condition of a sequence of predictable repeated postural perturbations. This is what happens during coordinated eye and head movements with gaze shifts that require whole body movements (71). The brain centers responsible for the anticipatory adjustments would be more than able of exploiting and properly integrating the visual and gravito-inertial inputs in order to fix the head with respect either to the space or to the platform as necessary.

### Limitations and Perspectives

We are aware that reading the text while balancing constitutes a dual-task, a cognitive and a postural one. The study was not designed, and did not contain control experiments, that might help disentangle the cognitive effect from the postural outcome. Indeed, it is not unlikely that diverting attention away from the postural task by reading the text (EO-TP and EO-TG) contributed to an overall limited head oscillation compared to EC (72). It has been suggested that concurrent cognitive (73) and active visual tasks (74) can be advantageous for (unperturbed) posture. High level processes such as expectation can modulate the impact of vision as well (30). In our case, continuously drawing attention away from the challenging postural condition per se may have favored the emergence of an easy head-moving-with-platform behavior. Suitable protocols can help address the issue.

We would also mention that the change in the balancing behavior produced by replacing the position of the text (moving with the platform vs. earth-fixed) in turn entrained a change in the visual flow. We are not in the position of discussing whether selective reweighting of the input from the visual field occurred in our study, because no *ad-hoc* experiment was made with changes in visual flow matching head displacement or with selective gating of the peripheral field. However, attention was focussed on the text for visual processing of foveal input (75, 76), as implicit consequence of the instruction. This occurred on a sustained basis, because the same target was maintained for the entire block of perturbation cycles, and reading was fluent, continuous and not interrupted by periods of e.g., looking at the environment. Under these circumstances, it would not be implausible that task-related visual maps would modulate the input from the peripheral visual field. It is known in fact that attention can modify gain control of the input from parafoveal target locations (77). Indeed, visual information is not stereotyped, but can be suppressed as a whole (38), but also partly [e.g., through salience maps, (78)]. These questions can be addressed by selective gating of the visual field during related experimental protocols.

The balance-challenging conditions employed here have constant amplitude (and constant frequency within each tested frequency) and are thus fully predictable. Under such conditions, feed-forward strategies are employed to generate anticipatory postural adjustments. These could be poorly dependent on visual or vestibular input or proprioception (17, 19–21, 45, 79), even if anticipation does not prevent consistent variability of the balancing behavior (46). We are not able to prefigure the effects of our present manipulation when inflow from the three sensory systems would become much more important as during conditions with unpredictable perturbations.

## Conclusion

Vision *per se* does not produce automatic head stabilization in space when the body is subjected to a continuous predictable sinusoidal horizontal translation of the support base. This is clearly shown by the large head displacements when subjects read a text fixed to, and moving with, the support base. Contrary to traditional interpretations, our results suggest that head stabilization may be revoked by the CNS as a mechanism to ensure a reliable reference for inertial guidance for postural control (80, 81), when necessary for accurate performance of a concurrent visual task. This finding is in line with the conclusion of pioneer investigations, performed under different balance conditions, showing that a supra-postural task influences postural control in an adaptive manner (26, 42, 82). In other words, vision controls the head position in order to accomplish the visual task optimally rather than to produce a necessary head and body stabilization in space.

The novel paradigm exploited here has the potential to significantly shift long-standing views on predictive balance control, since visual spatial attention modulates postural control in a so far undescribed way. Further investigations on the effect of attentional focus and cognitive tasks on postural control under equilibrium-challenging dynamic conditions are warranted (83, 84). Changing the visual reference during a rehabilitation treatment might be considered as a potential option for enhancing flexibility of coordination modes and multisensory integration processes in people with balance problems of various origin.

## Data Availability

The datasets generated for this study are available on request to the corresponding author.

## Ethics Statement

All subjects gave written informed consent to participate in the experiments, which were performed in accordance with the Declaration of Helsinki. The institutional ethics committee (Ethics Committee, Istituti Clinici Scientifici Maugeri IRCCS, approval number # 2257 CE) specifically approved the study.

## Author Contributions

SS contributed with data collection, data analysis, and drafted parts of the manuscript. AN contributed with data collection, data analysis, and read the final draft of the manuscript. MS contributed with project creation, data analysis, and wrote and edited the manuscript.

### Conflict of Interest Statement

The authors declare that the research was conducted in the absence of any commercial or financial relationships that could be construed as a potential conflict of interest.
